# When drug discovery meets web search: *Learning to Rank* for ligand-based virtual screening

**DOI:** 10.1186/s13321-015-0052-z

**Published:** 2015-02-13

**Authors:** Wei Zhang, Lijuan Ji, Yanan Chen, Kailin Tang, Haiping Wang, Ruixin Zhu, Wei Jia, Zhiwei Cao, Qi Liu

**Affiliations:** Department of Central Laboratory, Shanghai Tenth People’s Hospital, School of Life Sciences and Technology, Tongji University, Shanghai, China; Huai’an Second People’s Hospital affiliated to Xuzhou Medical College, Huai’an, China; R & D Information, AstraZeneca, Shanghai, China; Department of Computer Science, Hefei University of Technology, Hefei, 230009 China

**Keywords:** Learning to Rank, Virtual screening, Drug discovery, Data integration

## Abstract

**Background:**

The rapid increase in the emergence of novel chemical substances presents a substantial demands for more sophisticated computational methodologies for drug discovery. In this study, the idea of *Learning to Rank* in web search was presented in drug virtual screening, which has the following unique capabilities of 1). Applicable of identifying compounds on novel targets when there is not enough training data available for these targets, and 2). Integration of heterogeneous data when compound affinities are measured in different platforms.

**Results:**

A standard pipeline was designed to carry out *Learning to Rank* in virtual screening. Six *Learning to Rank* algorithms were investigated based on two public datasets collected from Binding Database and the newly-published Community Structure-Activity Resource benchmark dataset. The results have demonstrated that *Learning to rank* is an efficient computational strategy for drug virtual screening, particularly due to its novel use in cross-target virtual screening and heterogeneous data integration.

**Conclusions:**

To the best of our knowledge, we have introduced here the first application of *Learning to Rank* in virtual screening. The experiment workflow and algorithm assessment designed in this study will provide a standard protocol for other similar studies. All the datasets as well as the implementations of *Learning to Rank* algorithms are available at http://www.tongji.edu.cn/~qiliu/lor_vs.html.

**Graphical Abstract:**
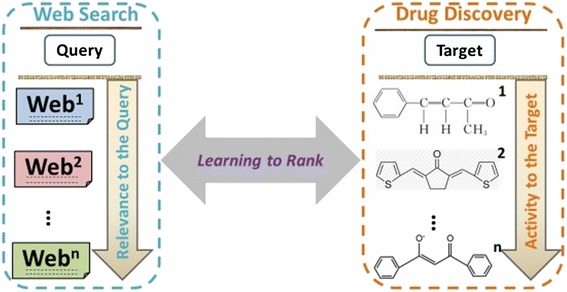
The analogy between web search and ligand-based drug discovery

## Background

**Figure 1:**
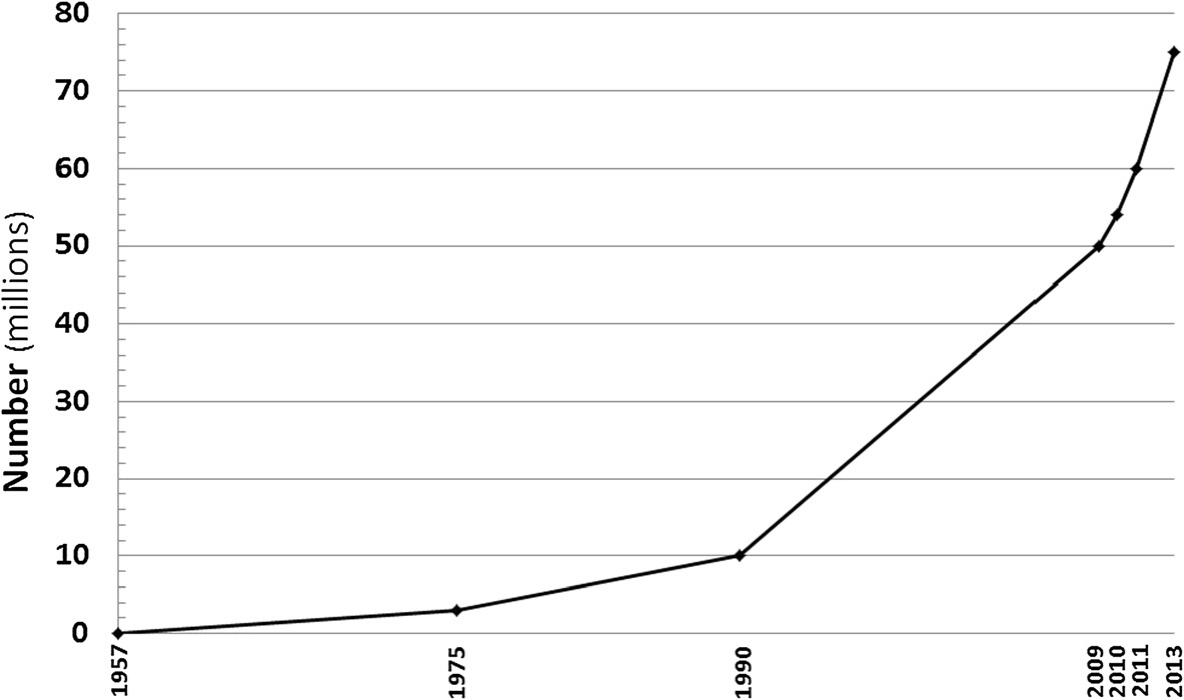
**Amount of CAS registry records of chemical substance.**

The cost of developing a new drug today is estimated to be over several billions with around more than ten years’ efforts. While a large portion of this cost results from the failed molecules, where the candidate chemical compounds are proven to be unsuitable for further development in preclinical and clinical testing [1]. With millions chemical structures available in the public library (Figure 1), more sophisticated and accurate computational screening approaches are highly demanded. Particularly, computational methods that “rank” chemical structures based on their likelihood of clinical success are useful for large-scale compounds screening. Such technologies, often termed as Virtual Screening (VS) [2,3] are used to focus on a small set of highly promising candidates for further experimental testing, leading to potentially huge time and cost savings.

Generally, the task of ligand-based VS is to output a ranking list of a set of molecules in terms of their binding affinities for a given drug target, so that the top-*k* molecules can be further examined through *in*-*vivo* or *in*-*vitro* test. The most basic technique utilized in VS is similarity search, which can be performed by firstly setting the target compound and then calculate the similarity between each compound and the target one. For this step, many different strategies of similarity measurements have been developed, including Cosine Coefficient, Euclidean Distance, Soergel Distance, Dice coefficient and Tanimoto coefficient [4]. Based on the similarity scores, the candidate compounds will be ranked and the top-*k* compounds can be selected for further investigation. Specially, VS can also be formulated as to learn a function *f* : *Structure* → *Activity* (*R*^*d*^ → *R*) based on a set of training compounds with known affinities for the target. The learned function can be used to predict the label (compound affinity) for any given molecules according to their structural features. Traditionally, this function can be learned as a regression or classification form, similar to the procedure of Quantitatively Structure Activity Relationship (QSAR) study [5].

Recently, a new emerging computational strategy called *Learning to Rank* (*LOR*) [6,7] that was firstly utilized in information retrieval field especially for the web search, has gained much attention. Web search and VS can be treated as a similar problem, seeking an analogous result where higher candidates (webs or compounds) should have higher relevance to the underlying target (query or protein). Taking this fundamental similarities into consideration, *LOR* should be a promising technique for solving VS problem; however, very few studies were performed in this area.

**Figure 2:**
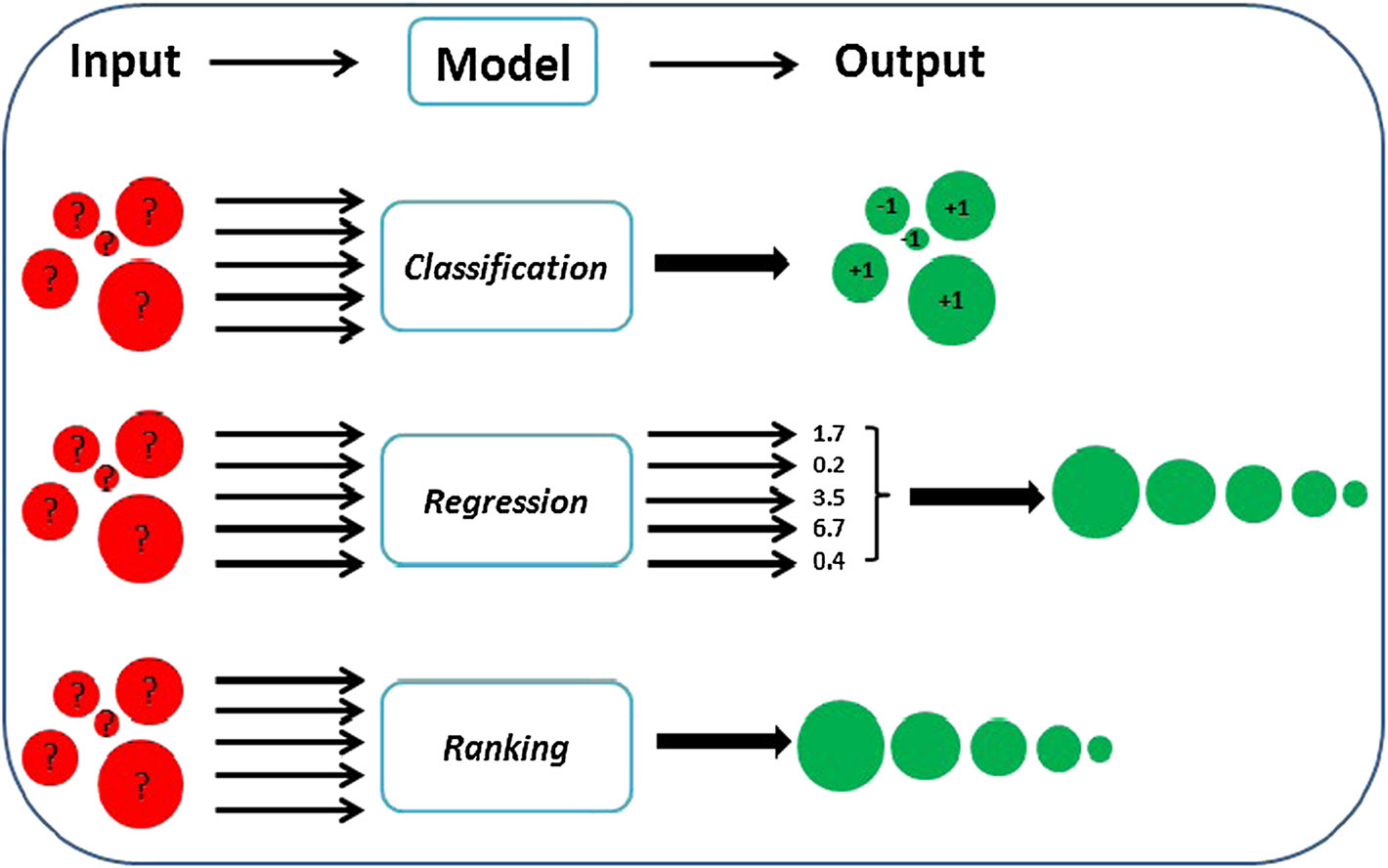
**Different computational schemas in virtual screening.**

The basic idea of *LOR* is to “learn” a rank function instead of traditional regression or classification function to predict the activity of candidate compounds for the query target. We see from limited literatures where *LOR* has been slightly touched on in drug discovery research. For examples, in 2009, Anne Mai Wassermann et al. utilized a Support Vector Machine (SVM)-based ranking method to distinguish compounds [8]; in 2010, Shivani Agarwal et al. introduced a bipartite ranking method on a relative small set of drug affinity data [1]; in the following year, Fabian Rathke et al. presented StructRank [9] which has shown competitive performance with traditional VS methods. Other applications of ranking in drug discovery include drug target fishing [10], drug descriptors selection [11] and chemical entity order analysis [12,13] etc. Although these works applied ranking techniques in VS, there is no systematic and benchmark study established for *LOR* in drug discovery so far, and the current methods were not generalized to cross-target screening. Basically, the goal of VS approaches is to learn a general ranking function which could be used for cross-target compound screening. It should focus on molecules with high binding affinities to the target while the predictive accuracy for the exact affinity labels is only of secondary interest [9]. Noted that traditional regression or classification model can also predict the different levels of the molecules of interest, this may not capture the intrinsic ranking order of the molecules [9]. As an illustration, for the traditional classification-based QSAR models, they are trained based on a set of molecules with known classification labels for a given target. It is clearly that the learned models only categorize the molecule activity into different known groups rather than ranking the molecule individually. For the traditional regression-based QSAR models, they are generally trained to minimize the squared error-based loss function for a given group of molecules, while equal models in terms of their mean squared error could give rise to completely different ranking results [9]. Therefore, the question arises whether the detour via regression or classification is necessary and whether the task can be addressed in a more straightforward way to directly derive the ranking function in VS (Figure 2) [9]. Given the aforementioned consideration, we proposed in this study the novel *LOR* model through learning a ranking function that focuses on the ranking relationship among all compounds rather than the exact activity or classification of each individual compound, which is inherently suitable in the identification of top-*k*-ranked compounds in VS.

Compared with traditional statistical learning based VS methods, *Learning to Rank* has the following two unique capabilities of (1). Applicable of extension to screen compounds on novel targets when there is not enough data available for these targets, and (2). Integration of heterogeneous data when compound affinities are measured in different platforms. Here, we have developed an integrated framework, which includes (1) a standard pipeline for *LOR* analysis in virtual drug screening, (2) comprehensive performance assessment for different *LOR* algorithms, and (3) public available testing benchmark data. In particular, the experimental workflow and algorithm assessments designed in this study will provide a standard protocol for other similar studies in drug discovery.

## Results and discussion

### Results of different testing strategies

*LOR* in VS aims to create a ranking function which could return the input compounds with a relevance descending order. In this study, six specific *LOR* models were comprehensively tested and compared for virtual drug screening. They are PRank [14], RankNet [15,16]_ENREF_13, RankBoost [15,17]_ENREF_15, SVMRank [18,19], AdaRank [15,20]_ENREF_18 and ListNet [15,21]_ENREF_19, which covers the three main categories of *LOR* (See *Methods*). Support Vector Regression (SVR) was set as the baseline VS method, and it was implemented and optimized with LibSVM [22]. A comprehensive testing pipeline was designed to compare the performance of six *LOR* models on the curated molecule affinity datasets. The testing datasets (Table 1 and 2) were collected from two public data sources, the Binding Database (BDB) and the 2012 benchmark dataset published by Community Structure-Activity Resource (CSAR). Four specific testing strategies were designed to achieve a comprehensive quantitatively performance evaluation of the models from different perspectives (See *Experimental*). Noted that there are various QSAR modeling based screening techniques while essentially they are learning based, thus only the typical SVR method was selected for comparison. The main purpose of this study is not to show the superiority of *LOR* to traditional methods, rather to present that *LOR* is an alternative option in VS and has its advantages to be extended for screening molecules on novel target as well as its utility in data integration. In the following section we will show the investigation results of different testing strategies and make the corresponding summaries respectively.

**Table 1:** **Curated bingding database dataset**

**Target**	*Ligand number*	**Target**	*Ligand number*	**Target**	*Ligand number*
**ADORA3**	*240*	**EPHX2**	*525*	**MK2**	*405*
**BDKRB2**	*155*	**FBPase**	*255*	**MMP**-**8**	*465*
**CB1**	*680*	**HMGCR**	*165*	**ORL1**	*270*
**CTSK**	*780*	**Itgαvβ3**	*220*	**PDE5**	*955*
**CCK1**	*430*	**JAK2**	*455*	**EP3**	*205*
**CHRM1**	*360*	**KIF11**	*295*	**SGLT2**	*515*
**CHRM3**	*430*	**LXR**-**beta**	*355*	**CYP17**	*205*
**TOP1**	*190*	**mTOR**	*585*	**ASC**	*190*

**Table 2:** **Curated CSAR dataset**

**Target**	**CDK2**	**CDK2**-**CyclinA**	**LPXC**	**Chk1**	**Erk2**	**Urokinase**
**Ligand number**	25	25	-	110	52	35
**Activity measurement**	Kd	Kd	-	pIC50	pKi	pKi
**Used**	No	No	No	YES	YES	YES

In the original CSAR dataset, LPXC has no compound affinity information, and the compound affinity associated with CDK2 and CDK2-CyclinA were measured with Kd value, which is a rough way to measure the affinity of combination rather than the exactly activity. These three targets were not selected in the final curated dataset.

It should be noted that in the following testing strategies Normalized Discounted Cumulative Gain (NDCG) was applied for the quantitatively comparison of different VS methods. NDCG was originally presented in information retrieval community to quantitatively measure the ranking results of instances based on its position in the ranking list. Basically in the ranking performance evaluation, we keep a grand-truth ranking list which is the molecule ranking for a given target based on their known efficacy. Then for different VS methods we obtain different predicted ranking lists based on different prediction models. These predicted ranking lists can be compared to the ground-truth ranking list to evaluate the VS performances respectively, as measured by the value of NDCG. Detailed information to calculate NDCG can be seen in *Methods.*

### Strategy I

**Figure 3:**
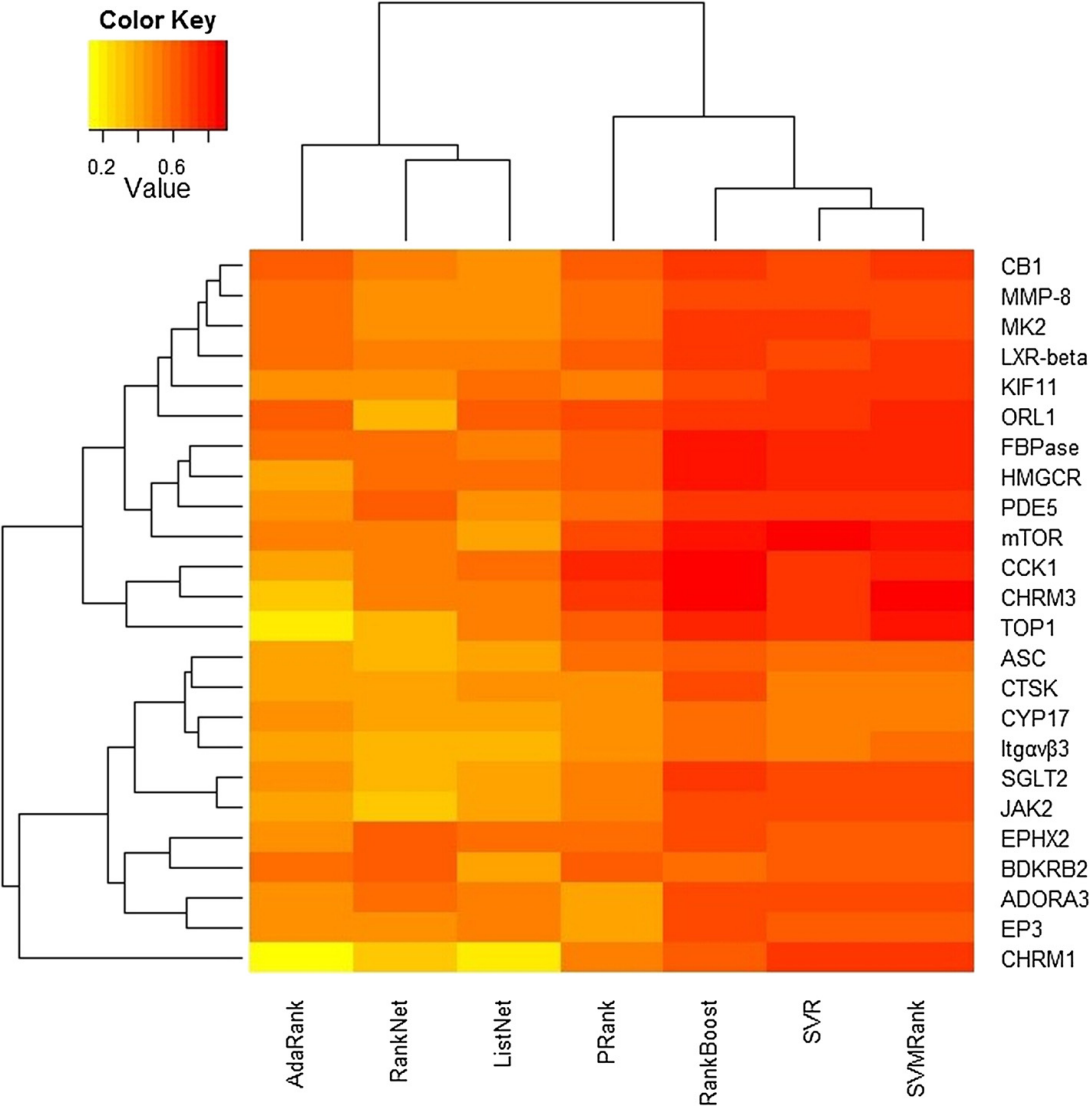
**NDCG**@**10 in Strategy I.**

This strategy was designed to compare *LOR* with the traditional SVR based VS techniques, and mimic the scenario that for a given target, there exited compounds with known affinities and they are trained to screen novel compounds. In this strategy, each protein target among the 24 curated targets from BDB and its associated compounds was treated as a task respectively. For each task, 5-fold cross validation was performed on six *LOR* models compared with SVR based method. The 5 times averaged NDCG value for each target among the 24 ones were calculated for quantitatively performance evaluation. As a result (Figure 3, Table 3), RankBoost and SVMRank performed the best among the six *LOR* models, and they are slightly better than SVR based method.

**Table 3:** **NDCG@10 of strategy I**

	**AdaRank**	**RankNet**	**ListNet**	**PRank**	**RankBoost**	**SVMRank**	**SVR**
**ADORA3**	0.4463	0.5885	0.5119	0.4032	0.6543	0.6446	*0.6815*
**BDKRB2**	0.5549	**0.6186**	0.4208	**0.6242**	0.5564	0.5917	*0.5935*
**CB1**	0.5913	0.4983	0.4586	0.6052	**0.6993**	**0.7026**	*0.6921*
**CTSK**	0.4225	0.3850	0.4741	0.4673	**0.6545**	**0.5253**	*0.5199*
**CCK1**	0.4122	0.5110	0.5704	**0.7661**	**0.8523**	**0.7673**	*0.7136*
**CHRM1**	0.1254	0.2978	0.1825	0.5366	0.6341	**0.7076**	*0.7068*
**CHRM3**	0.3295	0.5366	0.4880	**0.7282**	**0.9019**	**0.8738**	*0.7277*
**TOP1**	0.2076	0.3441	0.5005	0.6284	**0.7746**	**0.8101**	*0.7387*
**EPHX2**	0.4749	**0.5997**	0.5481	0.5506	**0.6604**	**0.6102**	*0.5913*
**FBPase**	0.5476	0.5420	0.5328	0.6281	**0.8081**	**0.7810**	*0.7710*
**HMGCR**	0.4078	0.5584	0.5475	0.6169	**0.8089**	**0.7956**	*0.7660*
**Itgαvβ3**	0.4168	0.3436	0.3555	0.4605	**0.5837**	**0.5399**	*0.5360*
**JAK2**	0.4208	0.3270	0.4184	0.5256	**0.6804**	**0.6653**	*0.6548*
**KIF11**	0.4682	0.4684	0.5724	0.5172	0.6912	**0.7267**	*0.7163*
**LXR**-**beta**	0.5828	0.5293	0.5009	0.6288	**0.7260**	**0.7104**	*0.6899*
**mTOR**	0.5204	0.5169	0.4038	0.6657	0.8334	0.8357	*0.8517*
**MK2**	0.5860	0.4398	0.4510	0.5909	**0.7299**	0.6945	*0.7272*
**MMP**-**8**	0.5792	0.4819	0.4843	0.5758	**0.6699**	**0.6841**	*0.6815*
**ORL1**	0.6082	0.3600	0.6024	0.6530	0.7270	**0.7656**	*0.7430*
**PDE5**	0.4877	0.6042	0.4628	0.5718	**0.7368**	**0.7237**	*0.7117*
**EP3**	0.4489	0.4484	0.5028	0.4054	**0.6504**	0.6292	*0.6306*
**SGLT2**	0.4619	0.3547	0.4285	0.5053	**0.7047**	0.6826	*0.6843*
**CYP17**	0.4829	0.4057	0.4001	0.4823	**0.5637**	0.4887	*0.5231*
**ASC**	0.4251	0.3584	0.4199	0.5630	**0.6243**	0.5629	*0.5813*

The bold number among each row indicates the best performance among all the methods in this row.

As a summary, SVMRank was the most efficient one among others. The superiority of SVMRank probably due to that such a ranking method inherits the maximum-margin characteristics of SVM. It transfers the ranking problem into a partial order pair classification problem, and utilizes the maximum margin optimization in SVM to derive the optimal ranking order. Therefore SVMRank obtains a robust and satisfied performance in *LOR* [6,7]. This result indicates that given proper optimization, the pair-wise based *LOR* model may serve as a suitable option for VS. Compared to traditional SVR-based VS, *LOR* could be served as an alternative option and achieves the acceptable performance in VS.

Taking accuracy and efficiency into consideration, SVMRank was selected for comparison in the following testing. It should be noted that in the following strategies, traditional SVR based method does not make sense, since there are either no training data existed for the specific target or the training data are combined from different measurements.

### Strategy II

This strategy was designed to investigate the performance of *LOR* to screen compounds on novel targets when there is no or few ligand affinity data available for these targets. In this case, traditional learning based VS techniques are not suitable here, since there are no or few available training datasets for the specific target. Specially, for the 24 protein curated from BDB, every 23 protein targets and their associated ligands data were combined together to act as the training dataset, and then tested on the left one target among the 24 ones. The testing procedure was also performed for 5 times on the 5 random divided parts of the compounds associated with the left target, respectively. Based on this strategy, the testing datasets in the strategy I and II were made to be identical for equally comparison purpose. The 5 times averaged NDCG value for each target among the 24 ones were calculated for quantitatively performance evaluation.

**Figure 4:**
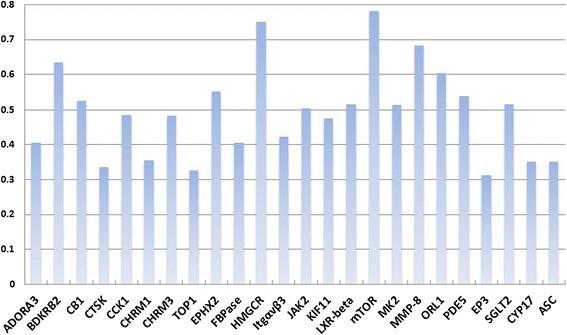
**NDCG**@**10 in Strategy II.**

In this test, SVMRank performed differently for different targets in this strategy (Figure 4). Generally, the performance is not as good as that in Strategy I but it is still acceptable, since this test was performed in a cross-target scenario. It can be seen that SVMRank made satisfied prediction on several specific targets, such as mTOR, HMGCR, MMP-8. Nevertheless, the unsatisfactory performance on other targets inspired us to investigate whether selecting phylogenetically related training target will benefit the testing results, which leads to the next strategy.

As a summary, SVMRank can be served as an efficient method for cross-target VS, and the performance can be improved when much more biological and pharmaceutical information are taking into considerations, as shown in the following.

### Strategy III

**Figure 5:**
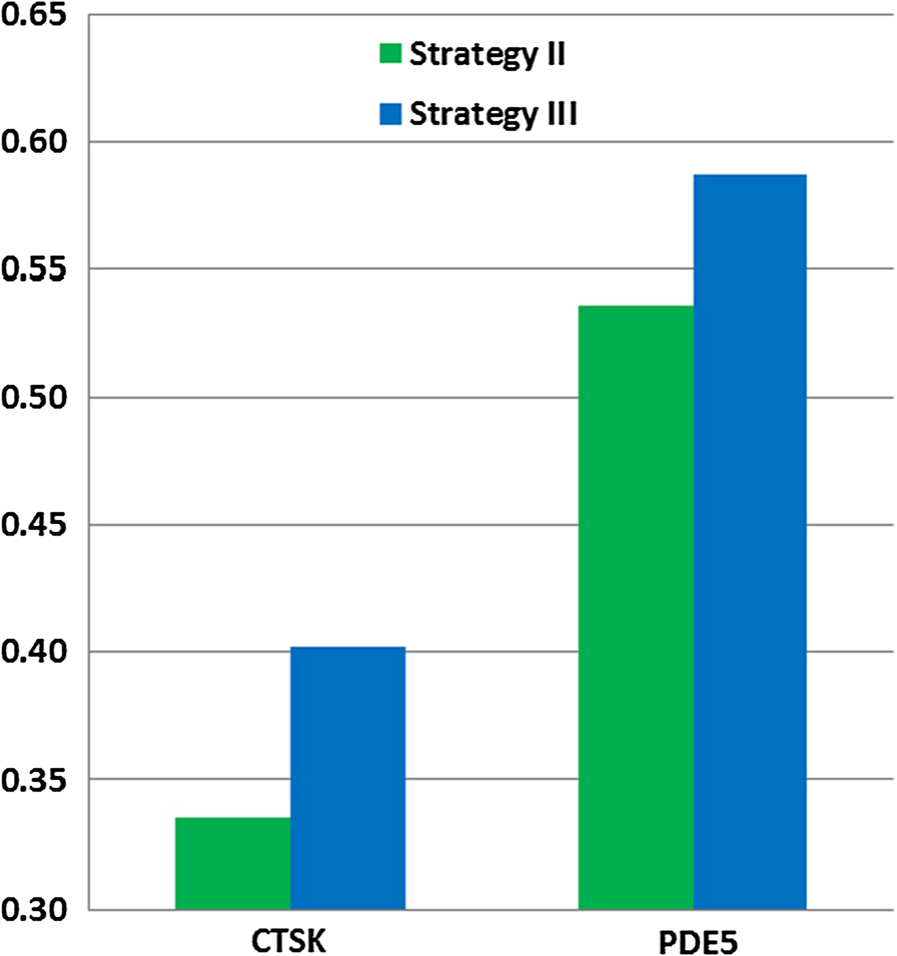
**NDCG**@**10 of CTSK and PDE5 in Strategy II and Strategy III.**

Compared to strategy II, in this test the training dataset was formed as the compounds data associated with the targets that belongs to the same family of the test protein target, to test the influence of protein phylogenetic feature in the prediction. In this strategy, among the original 24 targets, PDE5 and CTSK belong to two big protein families respectively. For each of these two targets, their remaining family members and the corresponding compound data in BDB were selected to form the training dataset (Table 4 and 5). This strategy was designed to check whether the training set formed from the same protein family would benefit the screening results on novel target under the *LOR* schema, since they are phylogenetically related. The testing datasets in the strategy II and III on proteins PDE5 and CTSK are made to be identical for equally comparison purpose. The NDCG value for the two targets PDE5 and CTSK were calculated for quantitatively performance evaluation. As shown in Figure 5, the final predictions for these two targets were improved substantially compared to those in Strategy II.

**Table 4:** **PDE family**

**PDE**	**PDE 1a**	**PDE 1b**	**PDE 1c**	**PDE 2a**	**PDE 3a**	**PDE 3b**	**PDE 4a**
*Ligand number*	*8*	*16*	*46*	*238*	*157*	*61*	*530*
**PDE**	**PDE 4b**	**PDE 4c**	**PDE 6a**	**PDE 6c**	**PDE 9a**	**PDE 10**	**PDE 11a**
*Ligand number*	*595*	*93*	*46*	*6*	*61*	*553*	*107*

**Table 5:** **Cathepsin family**

**CTS**	**CTS B**	**CTS D**	**CTS E**	**CTS F**	**CTS G**	**CTS H**	**CTS L**	**CTS S**	**CTS Z**
*Ligand number*	*519*	*847*	*40*	*28*	*228*	*17*	*651*	*1*,*206*	*6*

As a summary, the results in this strategy supported that, at least in our dataset, the selection of phylogenetically related targets and their associated compound affinity data in the training process may benefit the cross-target prediction to a certain extent. Serving as an efficient cross-target VS method, *LOR* still has the potential to improve its performance when extended useful information are considered.

### Strategy IV

By using SVMRank, this strategy was designed to test the performance of *LOR* to integrate heterogeneous data in VS. The rationale to design this strategy is to mimic the scenario that the compound affinity data maybe measured in different platforms or in different affinity criteria. For example, in the following test, the curated CSAR dataset was used and the compound affinities for different targets were measured in different affinity indicators as pIC50 or pKi respectively. Traditional virtual screening method cannot integrate such heterogeneous data directly. In this dataset, the compound affinity for target Chk1 is measured in pIC50, and that for targets Erk2 and Urokinase are measured in pKi. To test the performance of *LOR* for these targets, every 2 targets and their associated compound affinity data were taken as training data. The trained models were tested on the left one respectively and the corresponding NDCG values were calculated. It can be seen that the affinity measurement for training data and testing data in this procedure are inconsistent thus they are heterogeneous. As a results, performance on target Chk1 and Erk2 is fairly well, but it is unsatisfied on target Urokinase (Table 6). As it is reported that the directly combining of target and compound feature may lead to limited biological representation meaning [23], a different feature mapping was introduced, i.e., the *cross*-*term* [23], which was calculated as: *T*^147^ ⊗*C*^32^ (the Kronecker product of the two feature vector for target and compound, see *Feature representations of targets and proteins* in *Methods*), resulted into a new 4074 dimensional feature vector. Such a feature representation is reported to be more representative with enhanced prediction ability in protein-ligand interaction analysis [23]. From Table 6 it can be seen that SVMRank improved the prediction performances on the Top-10 candidates for all the 3 targets by using such new feature representation, even though the training data are heterogeneous and of limited amount. Particularly, the utility of *cross*-*term* feature mapping promoted the testing result on target Urokinase.

**Table 6:** **NDCG**@**10 in strategy IV**

	**Chk1**	**Erk2**	**Urokinase**
**NDCG**@**10 of normal feature mapping**	0.6562	0.7726	0.4876
**NDCG**@**10 of cross**-**term feature mapping**	**0.6821**	**0.7754**	**0.5967**

As a summary, the test results indicate that *LOR* may serve as a good choice for integration of various heterogeneous compound affinity data in VS, and the design of proper feature mapping in *LOR* will also influence the final ranking result. While the design of the efficient feature mapping method remains an open question in this field.

### Discussion on various VS methods based on multiple target information

Basically all the traditional regression or classification based models require that the training and testing data are i.i.d, and they cannot handle cross-target or cross-platform data integration. Although these methods can be directly performed, the results are not comparable since these methods are theoretically not suitable for cross-target or cross-platform scenario in VS. While for *LOR*, it is theoretically applicable for cross-target screening for the following reasons (1). In *LOR* model, it treated the target-compound pair as a whole instance. It does not require the distribution of the training compound data and testing compound data to be identical, thus it is inherently suitable for cross-target situations, and (2). It only considers the ranking orders of the instances for a specific target rather than their exact affinity values. In *LOR* for a specific target, especial in the use of the pair-wise *LOR*, it transfers the compound affinity data to the pair-wise partially order pairs and treats these new order pairs as the instances. Therefore although the compound affinities associated with the target may be measured in different platforms, it will have no influence on their transferred order pairs. While for traditional regression or classification based model it commonly treats all the compound data associated with different targets as a mixture dataset, thus their cross-platform effect should be taken into considerations.

**Figure 6:**
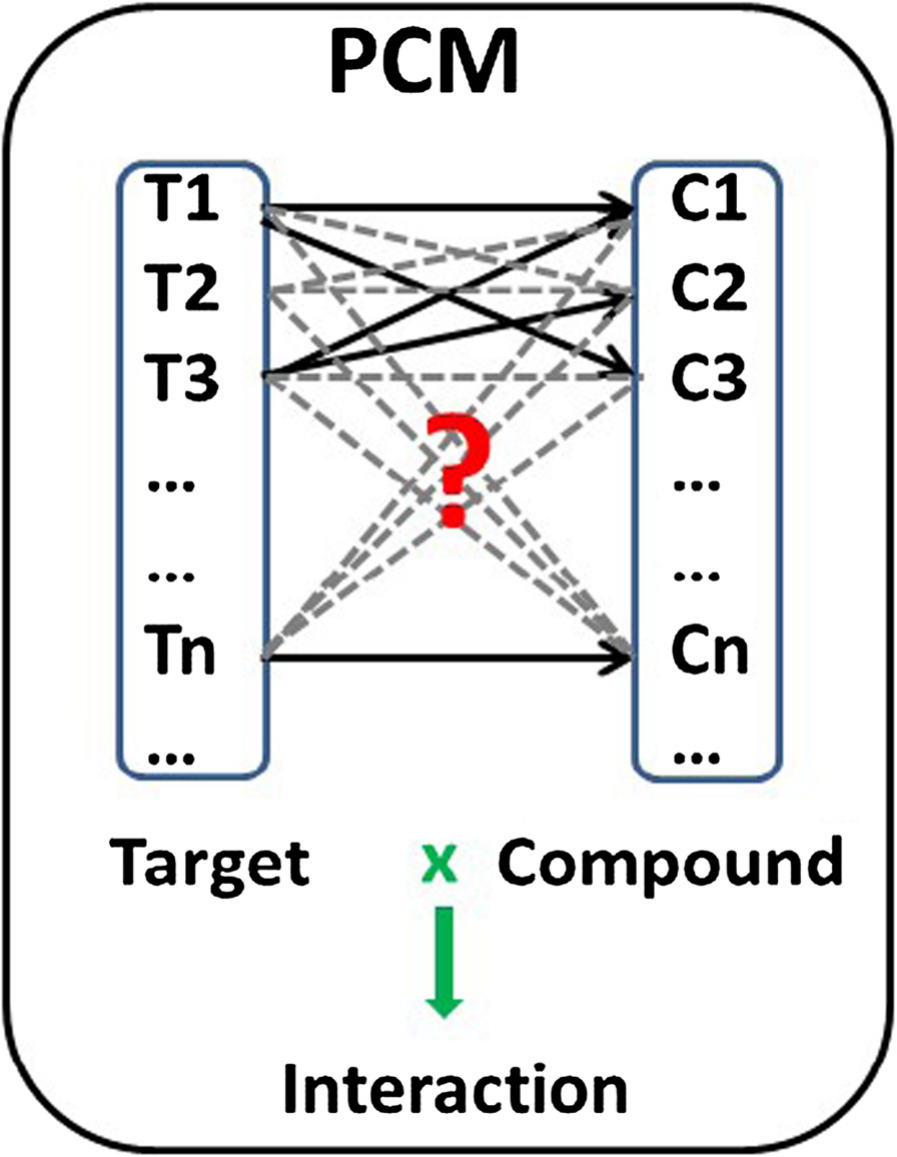
**Proteochemometric Modeling.**

*LOR* can be categorized to the idea of multi-targets based QSAR modeling for VS. Our group has previous tested other three multiple targets based QSAR schemas [24,25] such as multi-task learning based QSAR modeling [26], collaborative filtering based QSAR modeling [27] and Proteochemometric Modeling (PCM) [28,29]. Compared to traditional VS methods, essentially all these methods can be used to integrate multiple target information rather than the single one. All these models are constructed on both ligand and target similarity, and it can be regarded as an extension of conventional QSAR modeling to model the relationship between multiple compounds and targets simultaneously. For the multi-task learning based QSAR modeling and collaborative filtering based QSAR modeling, the target information is implicitly embedded in one computational schema and the target descriptor is not required to be calculated, while for *LOR* and PCM, they explicitly require the target information. From this point of view, PCM is intrinsically the most similar to *LOR* in QSAR modeling among others (Figure 6). However, in theory *LOR* directly aims at minimizing a ranking loss function rather than a classification or regression loss, thus inherently suitable for VS and integration of cross-platform data.

Another important issue for *LOR* is the proper design of feature function ∅( ) (See Methods). In current study we just combine the two feature vector for protein and compound in two sides directly to form the new feature vector or use the *cross*-*term* feature mapping. Compared to the directly feature combination from two sides, the cross-term feature mapping is more efficient. Although these two representations have their advantages of simplicity while their biological meanings are waiting to be elucidated. Another possible way to generate the feature is to define the target-compound interaction fingerprint as applied in our previous work [30]. Such kind of fingerprint is biologically much more meaningful while they are often not applicable for large-scale data since the generation of the fingerprint is time-consuming. We hoped that in the coming future more efficient and meaningful feature functions can be investigated.

## Experimental

### Testing pipeline

**Figure 7:**
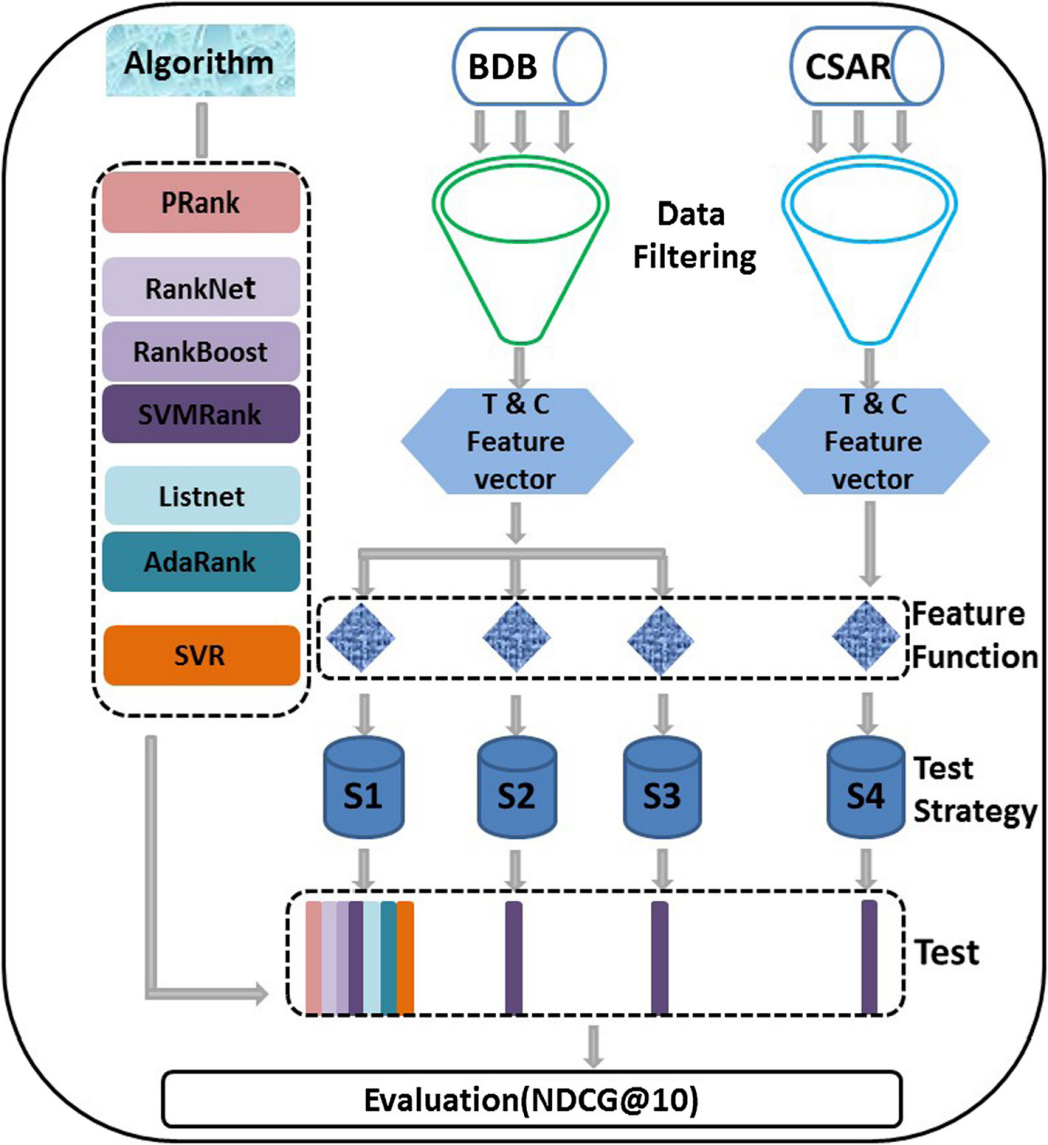
**Research workflow for** ***LOR.*** The datasets used in this study were curated from Binding Database and CSAR by well-designed filtering rules. The compounds and targets are represented in a specific feature vector respectively. With certain feature mapping function, the compound-target pair as a whole is transferred to a new feature vector. Based on four different testing strategies, the testing results on different VS algorithms are presented and evaluated quantitatively with NDCG@10. The color bars in the test frame indicate the corresponding algorithms investigated in the specific test strategies.

A comprehensive testing pipeline was designed to compare the performance of six *LOR* models on the curated molecule affinity datasets. There are mainly three points need to be addressed in this pipeline: (1). What is the performance of *LOR* compared with traditional SVR method, (2). What is the performance of *LOR* when it is extended to screen compounds on novel targets if there is no or few compound affinity data available for these targets, and (3). What is the performance of *LOR* to integrate heterogeneous data in VS when the compound affinities are measured in different platforms. The general pipeline designed in this study is shown in Figure 7. The brief introduction of the data and testing strategies are presented below the figure.

### Benchmark datasets generation

The testing datasets were collected from two public data sources, the Binding Database and the 2012 benchmark dataset published by CSAR. To make a relatively objective and balanced dataset, for the BDB, protein targets and their associated compound affinities data were selected based on the following criteria: (1). Only human protein targets are considered; (2) The redundancy of protein targets are eliminated; (3) The protein targets are selected to cover as many protein families as possible, and the proteins from the same family are avoid to be selected again as much as possible once other members in this family were selected; (4) To keep the data balanced, only targets with non-redundant ligands record number between 500 and 1,500 are considered; and (5) The affinity distribution of the compounds associated with a given target should be even. Taking pIC50 value as the affinity measurement, normally a compound is considered to be active if its pIC50 value is higher than 6 (pIC50 ≥ 6) [27], and inactive vise verse. The affinity was roughly graded into 5 categories as 0 (pIC50 < 6), 1 (6 ≤ pIC50 < 7), 2 (7 ≤ pIC50 < 8), 3 (8 ≤ pIC50 < 9), 4 (9 ≤ pIC50) according to reported literatures and we required that the associated compound affinity value should cover these 5 grades evenly. Those targets with associated compound affinities only have 0-grade and 1-grade, or the percentage of their highest grade data is fewer than 5% were also deleted. Based on these criteria, finally 24 proteins associated with 9,330 compounds were curated (Table 1). These data will be used in the former three testing strategies in the pipeline.

The second dataset is curated from the published 2012 CSAR benchmark dataset, which includes six protein targets and several of them have associated compound affinity information, while measured in different standards, including pIC50 and pKi value. In this dataset, only target Chk1, Erk2 and Urokinase with associated compound affinity data were tested in the fourth strategy in the pipeline (Table 2).

## Conclusions

In this work, a comprehensive investigation on *LOR* was performed on benchmark datasets and the experiment workflow and algorithm assessment was presented. The results indicate that *LOR*, especially the pair-wise methods like SVMRank, can be served as an alternative option for VS compared with traditional methods. Furthermore, *LOR* has its inherent advantages to be extended for screening molecules of novel target as well as its utility in data integration. For a certain novel protein target, no matter whether its associated known ligand affinity information existed or not, *LOR* can return a satisfied ranking result. It is also theoretically suitable to rank the compounds based on the training data measured in different platforms. In addition, several future work directions on *LOR* would be: (1) The integration of multiple feature representations of the target as well as the compound using other descriptors or profiles. The high-dimensional pharmoco-genomics information from CMAP [31,32]_ENREF_30 and PubChem BioAssay data [33,34] can be extensively investigated. The multi-view learning [35] based methodology can be investigated to integrate different representations to present the comprehensive target and compound description and similarity calculation; (2) The transfer learning [36] based methodology is needed in VS for the study of “cross-target knowledge transfer” to leverage the information of large-scale of target and compound data.

## Methods

### *LOR* model in VS

*LOR* in VS aims to create a ranking function which could return the input compounds with a relevance descending affinity order for the target. Traditionally, the similarity based ranking model in VS is constructed by purely similarity-based or regression/classification-based model. In *LOR* framework, we often learn a ranking function *f*(*T*, *C*), which is trained by minimize a ranking loss function on a set of compound *C*_*ij*_ (i = 1,2, …, m) for a given set of targets (*T*_1_, *T*_2_, … *T*_*m*_) [37]. Different from the traditional machine learning model for single target, the learned function has the generalized ability for novel data prediction. This means that for a novel target *T*_*m* + 1_ that is not seen in the previous training dataset, as long as it can be explicitly represented in the correspondence feature space, the system can also rank the compounds on this target.

**Figure 8:**
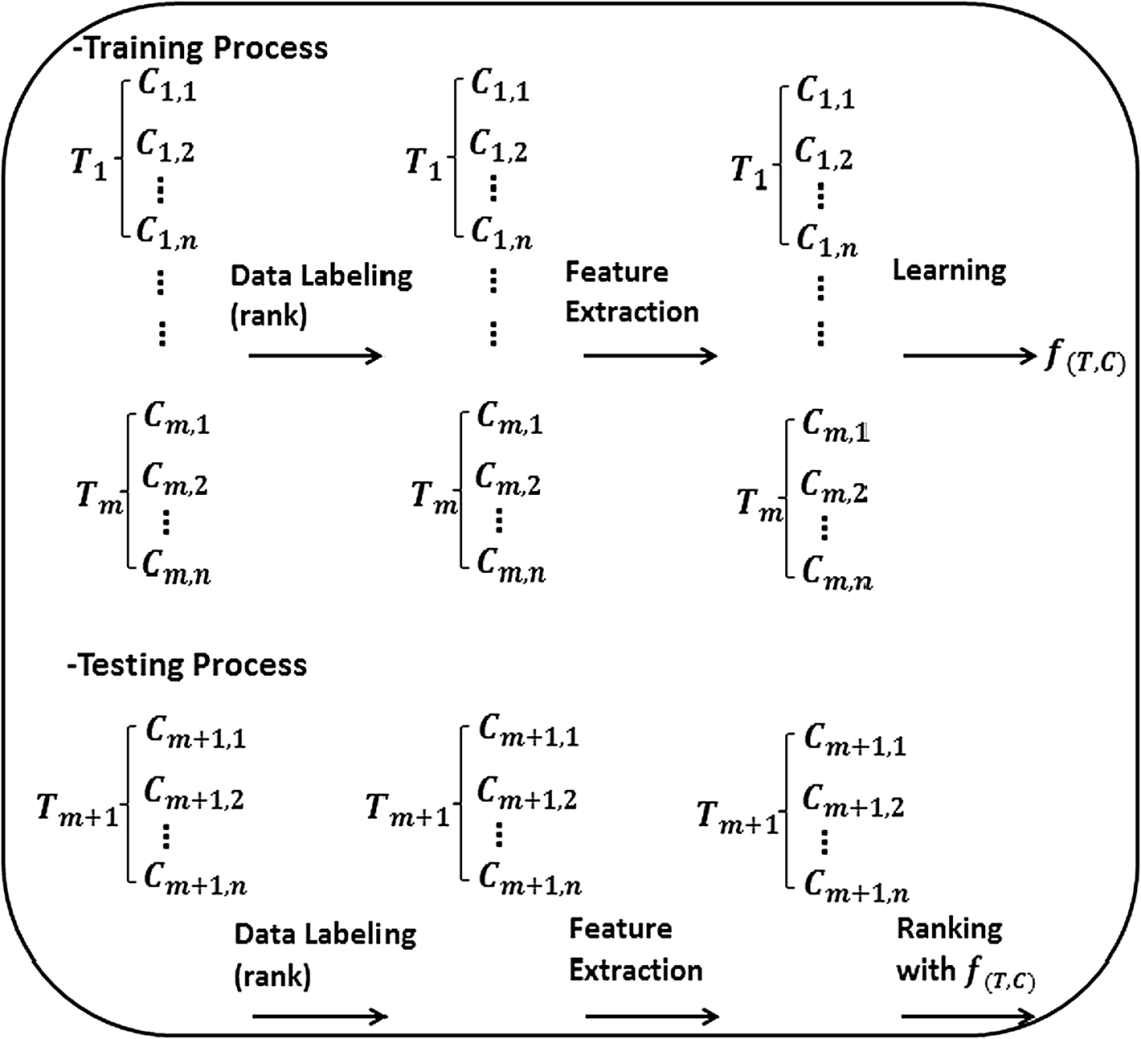
**Illustration of training and testing in** ***LOR.***

The specific *LOR* procedure is analogue to the traditional training and testing procedure in QSAR modeling. In *LOR*, the training data contains given targets and compounds. Each target is associated with a number of compounds. While the difference between *LOR* and traditional QSAR model lies that the *LOR* model often involve multiple targets rather than one single target. The relevance of the compounds with respect to the target is known, measured as the compound affinity either in a categorical label (High, medium, low etc.) or in a numerical value (IC50, EC50). Supposing that for a given target *T*_*i*_, (*T*_*i*_, *C*_*ij*_) is used to represent the target and its associated compound information, then a feature vector *x*_*ij*_ = ∅(*T*_*i*_, *C*_*ij*_) is created for each target-compound pair (*T*_*i*_, *C*_*ij*_), where ∅ ( ) denotes the feature function. In the training procedure, the aim of *LOR* is to train a local ranking model *f*(*T*, *C*) = *f*(*x*) that can assign a ranking score to a given target-compound pair *T* and *C*, with the feature vector *x* representing the whole target-compound pair [37]. In the procedure of testing, given a novel target *T*_*m* + 1_ which is not seen in the previous screening, the ranking function *f* can assign scores to the compounds. This can be achieved by taken the novel target *T*_*m* + 1_ with its associated compound *C*_*m* + 1,*j*_ as a pair (*T*_*m* + 1_, *C*_*m* + 1,*j*_). And then the pair can also be represented in a feature vector based on the feature mapping function *x*_*ij*_ = ∅(*T*_*m* + 1_, *C*_*m* + 1,*j*_). Using the trained model based other target pairs, the ranking position of *C*_*m* + 1,*j*_ to *T*_*m* + 1_ can be predicted, finally the molecule ranking list for this novel target can be obtained (Figure 8).

Compared to traditional QSAR modeling, *LOR* is different in that it focus on multiple targets rather than single target. *LOR* uses a bunch of targets with their associated compounds to train a generalized prediction model and makes prediction on the other targets (Figure 8). Therefore *LOR* is suitable for the cross-target screening. Such an extended ranking ability for the new target cannot be achieved with the traditional classification or regression model in VS [9].

**Figure 9:**
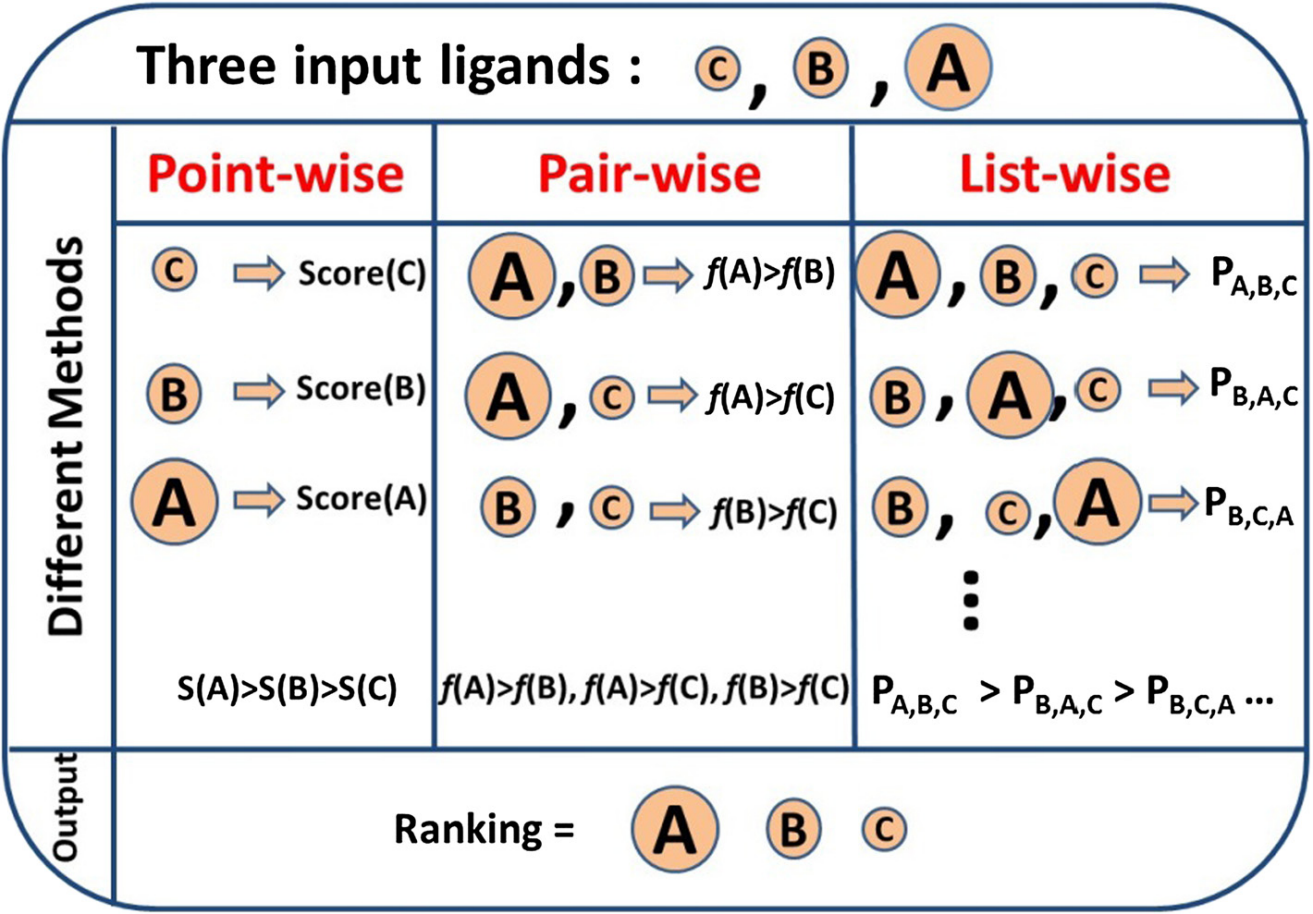
**Three different approaches of** ***LOR.***

Based on the distinct forms of input instance organization, generally there are three different approaches to realize *LOR*, and can be categorized into three types: point-wise, pair-wise and list-wise (Table 7, Figure 9). The point-wise and pair-wise approaches transform the ranking problem into classification, regression, or ordinal classification. The list-wise approach takes ranking lists of objects as instances in learning and learns the ranking model based on ranking lists. Detailed information can be referred in the literature [38].

**Table 7:** **6** ***LOR*** **algorithms**

**Approach**	**Algorithm**	**Reference**
**Point**-**wise**	PRank	[14]
**Pair**-**wise**	RankNet	[16]
	RankBoost	[17]
	SVMRank	[18,19]
**List**-**wis**	AdaRank	[20]
	ListNet	[21]

### Feature representations of targets and proteins

As aforementioned, in *LOR* framework, for a given target-compound pair (*T*_*i*_, *C*_*ij*_) a feature vector *C*_*ij*_ = ∅(*T*_*i*_, *C*_*ij*_) should be defined, where ∅( ) denotes the feature function. In this study, for ligands, the widely used General Descriptor (GD, 32 bit) is employed to represent the ligand in a 32-dimensional feature vector. GD measures a compound through four aspects, van der Waals surface area, log P (octanol/water), molar refractivity and partial charge [39]. For protein targets, they were depicted through CTD (Composition, Transition, Distribution) feature, which represents the amino acid distribution patterns of a specific structural or physicochemical property along a protein or peptide sequence. The protein target is represented in 147-dimension vector by the CTD feature. In this study, GD was calculated through the software Molecular Operating Environment (MOE, C.C.G., Inc. Molecular Operation Environment, 2008.10; Montreal, Quebec, Canada, 2008) and protein CTD feature was calculated by PROFEAT [40].

After representing target and compound respectively, the chosen of ∅( ) is important for the performance of *LOR*. In strategy I, II and III, the protein feature and compound feature were combined in two sides directly to form the new feature vector (totally 179-dimension). In strategy IV, the cross-term feature mapping function was also used to generate the new feature vector for target-compound pair representation. While the possibility of defining other forms of ∅( ) was discussed in *Results and Discussion*.

### Performance measurement

In order to quantitatively evaluate the VS performance under the *LOR* schema, Normalized Discounted Cumulative Gain (NDCG) was applied in evaluation. NDCG was originally presented in information retrieval community to measure the ranking results of instances based on its position in the ranking list. Specifically, assuming $\overline{y}$ is ideal ranking and *ŷ* is the predicted ranking, for the top-*k* in the predicted ranking list, NDCG [8] is calculated as following:

1 $$ \mathrm{NDCG}@k=\frac{\mathrm{DCG}@k\left(\widehat{y}\right)}{\ \mathrm{D}\mathrm{C}\mathrm{G}@k\left(\overline{y}\right)} $$

2 $$ \mathrm{D}\mathrm{C}\mathrm{G}@k={\displaystyle {\sum}_{r=1}^k\frac{2^{y(r)}-1}{ \log {}_2\left(1+r\right)}} $$

Where *y*^(*r*)^ is the rank label of the compound at *r*-th position in the ranking list.

Noted that if the predicted ranking is exactly the same as the ground truth, the NDCG value will be 1.0. This measurement can be used for the evaluation of *LOR* results compared to traditional regression or classification based performance measurements such as RMSE and accuracy etc. Also we noticed that there are some other ranking performance evaluations like *ERR* [41], *MAP* [42] etc., while they are not intuitionistic as NDCG does.

It also be noted that in this study, only the top-10 ranking results were evaluated with NDCG value, denoted as NDCG@10. This is a very strict evaluation criteria since the ideal ranking list can only be achieved when the top-10 known candidates were successfully predicted.
